# Knowledge, Attitudes, and Prevention Practices of Drug Resistant Tuberculosis in the Eastern Cape Province, South Africa

**DOI:** 10.1155/2019/8978021

**Published:** 2019-11-25

**Authors:** Thanduxolo Elford Fana, Edwin Ijeoma, Lizo Sotana

**Affiliations:** ^1^Centre for Health Policy, School of Public Health, University of the Witwatersrand, Johannesburg, South Africa; ^2^School of Government and Public Administration, University of Fort Hare, Bhisho, Eastern Cape, South Africa; ^3^School of Social Science and Humanities, University of Fort Hare, East London, Eastern Cape, South Africa

## Abstract

The aim of this study was to assess community members' knowledge and awareness levels, attitudes, and practices of Drug Resistant Tuberculosis. A quantitative descriptive cross sectional study was carried out in the Eastern Cape Province of South Africa. The sample size consisted of four hundred (400) respondents aged 18 years and above on their last birthday who were purposively and conveniently selected from Port Elizabeth area in the Nelson Mandela Municipality. Data were collected using close-ended questions, which were administered by the researcher and two research assistants to the selected respondents. Data were analysed using descriptive statistics. The results of this study show poor knowledge and awareness levels, unfavourable attitudes, but good prevention practices of Drug Resistant Tuberculosis among Port Elizabeth community members. This study also found a statistically significant association between knowledge and attitudes (*p* value = <0.001), and no statistically significant association between knowledge and practices and attitude and practices, respectively (*p* values = 0.120 and 0.136). The study also revealed low literacy levels, inadequate information, misconceptions and erroneous beliefs about causes, transmission, prevention, treatment, and management of Drug Resistant Tuberculosis among the respondents. This study also highlighted the use and existence of dual healthcare system (traditional spiritual and western).The study found that the main source of Drug Resistant TB information was radio and television among the majority of research respondents. It is recommended that in future health education interventions and awareness campaigns need to be intensified in the area so that misconceptions and erroneous beliefs that exist in society can be addressed. It is also recommended that training programs that are culturally sensitive should be developed and delivered taking into account different languages and literacy levels that exist in society. Such education interventions should be facilitated in collaboration with people living with Drug Resistant Tuberculosis. A multidisciplinary approach should be fostered and collaborations with spiritual healers and various congregational leaders, traditional health practitioners, community leaders, and government leaders in the health sector should be promoted in order to deal with Drug Resistant Tuberculosis. It is also recommended that a similar study be conducted using a qualitative research approach in urban and rural areas of the Eastern Cape. Lastly, assessment of knowledge, attitudes, and practices of spiritual and traditional healers with regard to Drug Resistant Tuberculosis should be conducted as they can influence health-seeking behaviour.

## 1. Introduction

Tuberculosis (TB) is caused by Mycobacterium tuberculosis. TB is an infectious deadly contagious but preventable disease. TB is regarded as a cause of ill health among millions of people around the globe, and the leading causes for death after Human Immunodeficiency Virus and Acquired Immunodeficiency Syndrome (HIV and AIDS) [1]. The high incidence of TB is a cause for concern and a huge threat to public health globally [2]. The delays in case detection, diagnosis, health seeking, and nonadherence to treatment are some of the reason for the high TB burden globally [3]. According to the World Health Organization (WHO) it was estimated that 10.4 million of new TB cases in the world and 10% was living with HIV. 95% were adults of whom 65% were males 74% of these people live in Africa and almost 64% of the total were from seven countries (India, Indonesia, Philippines, Russia, Pakistan Nigeria, and South Africa) [4].

The emergence of Multi Drug Resistant Tuberculosis (MDR-TB) further poses a more serious threat to control of TB worldwide and the development agenda especially in low medium income countries as it affects mainly the economically active population. In 2016, there was an estimated 600,000 new cases of Rifampicin Resistant Tuberculosis (RR-TB) globally of which 490,000 had MDR-TB. About 47% of the RR-TB cases lived in India, China, and Russia. 129,689 people were notified as having started MDR-TB treatment in 2016 [4]. In SA, the initial outbreak of the Drug Resistant TB was first identified in 2005 at the Church of Scotland Hospital in KwaZulu-Natal, Tugela Ferry. The mortality rate of 98% when it was identified [5]. In SA, KwaZulu-Natal (KZN) is the Province with the highest number of people infected with Multi Drug Resistant-TB, whereas Western Cape (WC) and the Eastern Cape (EC) are lying in the second and the third spot with regard to the number of people infected with MultiDrug Resistant-TB [6, 7]. Port Elizabeth (PE) in the Nelson Mandela Municipality (NMM) is one major city in the EC, South Africa. In 2011, TB was declared a crisis in the NMM and an announcement was made in the EP Herald that war against TB was lost in the NMM [8]. One in 100 people is infected with TB in the NMBM. Secondly, 90% of those diagnosed with TB are also coinfected with HIV and or AIDS. NMBM has also recorded the second highest rates of HIV deaths after Mangaung (185.9 per 100,000) with (123.4 per 100,000 (Health-e, 2018). NMBM has a 3rd highest rate of TB treatment defaulters and ranks among the ten worst metros in the country for deaths caused TB, TB cure and treatment success rate [9]. Therefore, it is therefore clear that TB is a major public health challenge in SA, NMM.

Previous interventions to control TB were largely biomedical. Research evidence shows biomedical interventions alone are not enough to curb the spread TB and the emerging Drug Resistant TB strains. Research indicates that changes in socio economic status and improvements in knowledge and attitudes strengthen TB prevention and control. The WHO has also included education interventions as one of its priorities [10]. Despite the growing interest in socio economic aspects of this disease, there is paucity of research on communities knowledge, attitudes and prevention practices of Drug Resistant TB. Poverty and lack of knowledge are regarded as factors that increase the risk of exposure among community members [11]. There is also an association between negative attitudes and low knowledge about a disease [15]. Lack of financial resources, poor knowledge, erroneous beliefs, and misconceptions regarding causes, mode of transmission, symptoms and treatment, does not only affect health seeking behaviour [11], but it promotes the use of spiritual, faith healers and traditional healthcare practitioners over biomedical methods. These approaches contribute to poor treatment adherence and delays in diagnosis and exacerbate spread of TB among the healthy community members [13]. Fear of social isolation, prejudice, stigmatization, and discrimination by family healthcare workers and community members also hinder TB prevention and control as they lead to treatment default, abandonment of treatment by those already diagnosed and nondisclosure of health status and delays in health seeking and diagnosis by community members [14].

The emergence of Drug Resistant TB, high mortality rate despite biomedical interventions that seek to mitigate and curb its spread makes it compelling to undertake this study. Assessment of community members' knowledge, attitudes and prevention practices about this deadly, highly contagious but preventable disease, is essential to inform planning, implementation and evaluation of advocacy, communication, and community mobilisation in the Nelson Mandela Municipality, Eastern Cape Province of South Africa.

## 2. Theoretical Framework

In this study, the researcher has chosen to use Knowledge, Attitude, Behaviour, and Practices (KABP) methodology. KABP is not a theoretical framework or philosophical paradigm, but a method used by public health researchers to assess community's understanding and response to a disease. KABP studies are highly focused evaluations that are used to measure changes in human knowledge, attitudes, and practices in relation to disease prevention. KABP studies tell us what people know about certain things, how they feel, and how they behave. In this study, only three aspect of the KABP methodology was considered and that is, knowledge, attitudes, and practices (KAP) of community members about Drug Resistant-TB [15]. This study emerges because of the observed gap in literature and theory with regard to Drug Resistant-TB among community members in Port Elizabeth in the Nelson Mandela Municipality. This study assesses Drug Resistant TB knowledge, attitudes, and prevention practices among the residents of Port Elizabeth in the NMM in the Eastern Cape Province of South Africa.

## 3. Research Methodology and Design

### 3.1. The Study Design

In this cross-sectional study, a descriptive, nonexperimental research design with a quantitative approach was used to investigate knowledge and awareness levels, attitudes, and prevention practices of Drug Resistant TB among residents of Port Elizabeth in the Nelson Mandela Municipality, Eastern Cape Province of South Africa.

### 3.2. The Study Setting

**Figure 1:**
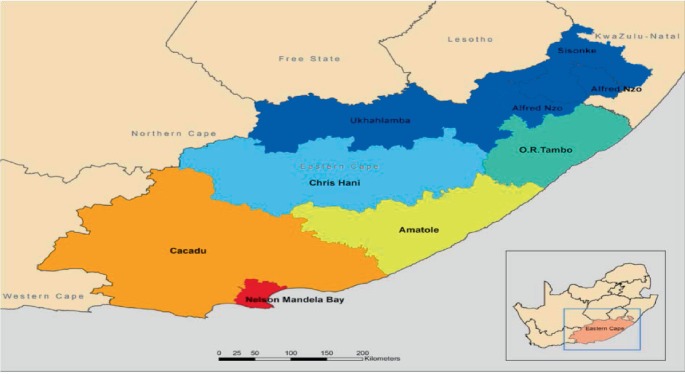
Eastern Cape district municipalities. Fana, Ijeoma and Eyles, 2018.

Figure 1 shows district municipalities in the Eastern Cape. Nelson Mandela Municipality is the third largest and most dense populated district in the Eastern Cape Province, with a population of about just over one million. The Nelson Mandela Bay Municipality has 60 wards and is made up of Port Elizabeth (48), Uitenhage and Despatch (12). The unemployment rate is in the NMBM is 36%, 45%, 3% Eastern Cape Province and 36.8% nationally. Female-headed households are at about 41% and it is in Port Elizabeth, Kwa-Zakhele in the Nelson Mandela Municipality where 98% of 580 households used bucket system and that 81% of them used paraffin as their source of energy [1,16].

### 3.3. Study Participants and Sampling

The study participants were residents of Port Elizabeth in the Nelson Mandela Bay Municipality who were 18 years old and above. The sample size was 422. It was calculated using the population of Nelson Mandela Bay Municipality 1,152,115 and statistical variables of 95% (CI), 5% significance interval and then factoring in a 10% nonresponse rate (384 + 38 = 422). In this study, multistage sampling was used to select the research respondents. The sampling strategy was purposive at one level, when only 48 Port Elizabeth wards from the list of the 60 wards in the Nelson Mandela Bay Municipality were selected for the study purpose. It was also random at another level, when every 5th ward was selected from the list of the 48 Port Elizabeth wards until 10 wards were selected for the purpose of this study. It was also purposive at another level, when the 18 years old and above research respondents were selected. It was also accidental or convenient at another level when data were collected in the 10 wards in those residential areas that were more accessible and closer to the researchers. 42 respondents were selected to participate in this study from each of the ten wards. Lastly, it was also accidental or convenient when data were collected from anyone that met the inclusion criteria, gave consent and was available in the house at the time of data collection.

### 3.4. Inclusion and Exclusion Criteria

The study included community members who were 18 years and above on their last birthday and gave consent for participation. All those who below 18 years of age, on their last birthday and had severe health problems with cognitive impairments were excluded from this study.

### 3.5. Research Instrument and Pilot Study

The researcher developed an English language questionnaire with the assistance of the research supervisor, health educator and a MDR-TB specialist after having conducted an extensive literature review. The researcher collected data with the assistance of two research assistants using these structured questionnaires. The first part of the questionnaire dealt with the socio-demographic details of the participants. The second part dealt with knowledge, attitudes, practices, and beliefs about the risk, severity, causes, transmission mode and prevention methods, diagnosis, treatment, and management of Drug Resistant Tuberculosis. The ratings were as follows: strongly agree 1, agree 2, disagree 3 and strongly disagree 4. The scale of 1–4 was used in this study in order to prevent the respondents from choosing a neutral answer.

A pilot study was conducted in two wards in the Despatch/Uitenhage area to test the feasibility of the study, and that included the methodology, administration procedures and the research instrument in order to check for inaccuracies and ambiguity. A sample of 63 individuals (15%) of the initial planned sample of 422 of the main study was included in the pilot study. These participants were excluded from the actual study. The validity of the research instrument was checked through inter-ratter assessment of its usefulness in collecting the required information from the respondents. Adjustments were made on time required to complete the questionnaires. In order to assess participant's knowledge, attitudes, and prevention practices of Drug Resistant TB, the respondents were asked to tick a response that represented their knowledge about the disease. The ratings were as follows: strongly agree and agree was made1 and disagree and strongly disagree was made 2. All correct answers yielded 1 point and incorrect opinions and missing data yielded no points. The responses were added together in order to yield scores that ranged between 0 and 20 for knowledge, 0–15 for attitudes and practices. High scores were an indication of high knowledge, good or favourable attitudes and practices of Drug Resistant Tuberculosis. A score 0–13 was categorised to be a poor knowledge score and a score from 14 to 20 was categorised as a good knowledge score. A score of 11–15 was coded as good or favourable, while a score of 0–10 was coded as poor or unfavourable for attitudes. A score of 11–15 was coded as good, while a score of 0–10 was coded as poor for practices.

### 3.6. Data Analysis

Data collected from the field study were checked for completion, cleaned, and entered into Microsoft Excel 2013, spreadsheet. Data were exported from Excel spreadsheet to SPSS® statistical software due to its flexibility and excellent capacity for labelling variables. Pearson's Chi square was done to establish relation between knowledge attitudes and practices, and a *p* value of = <0.001 was considered statistically significant. The results were presented using descriptive summary measures such as frequency, percentages, mean, standard deviation, and standard error. Frequency tables and graphs were used to show the pattern of participants' responses.

## 4. Research Results

### 4.1. The Socio-Demographic Characteristics of the Participants.

Out of the total sample size of 422, 400 individuals gave consent and completely answered the questionnaires making the response rate in this study 95%. There were 190 (47.50%) males and 210 (52.50%) females, ranging from 18 to 68 years (38 ± 12.2 years). The majority of the respondents were Africans (67.50%) and unemployed (62.50%). More than half of the respondents were females (52.50%) and single (54.50%).The results also show that the majority (58%) of the participants earned R 4 999 ($357) and less per month. More than a quarter of the respondents had primary education (37.50%) and tertiary education (30.75%) [1] (Table 1).

### 4.2. Participant's Sources of Drug Resistant Tuberculosis Information

Table 2 shows that more than a quarter respondents revealed that television and radio (34.25%) and television radio and internet (30.25%) were their main sources of Drug Resistant TB information, whereas, only (14%) mentioned HCW and teachers as their source of Drug Resistant TB information [1].

### 4.3. Participant's Knowledge of Drug Resistant Tuberculosis

**Figure 2:**
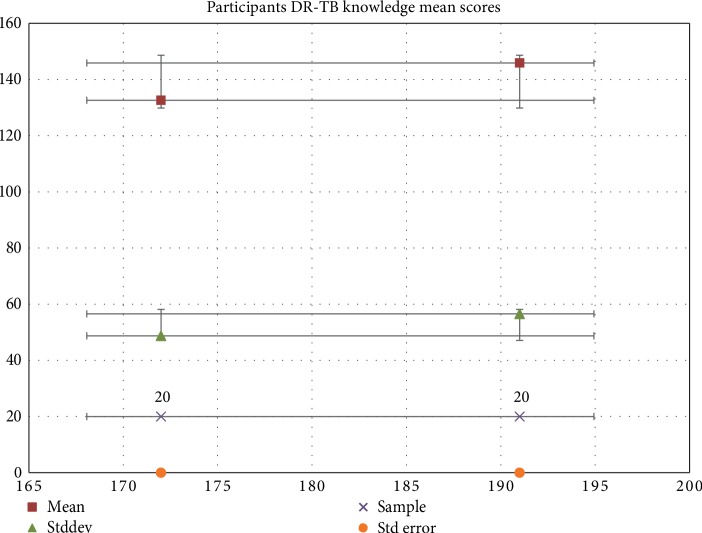
Graphic distribution of respondent's DR-TB knowledge mean scores.

Data in Figure 2 shows that on female had a higher Drug Resistant Tuberculosis mean score (145, 9) compared to males (132, 6). The results also indicated that there is a 95% chance that the population mean for females is within 12.646 standard error of the mean (145, 9) compared to 10.896 standard error of the mean 132, 6 of the males.

### 4.4. Participant's Perceptions and Attitudes about Drug Resistant TB

Table 3 provides information related to respondents perceptions and attitudes towards Drug Resistant TB. The results obtained from this study showed that the majority respondents believed that the community will avoid them but remain friendly, 23% indicated that the community would reject them, while 27% indicated that the community would be supportive if they are infected with Drug Resistant TB.

The study also revealed that if diagnosed with Drug Resistant TB the majority of the respondents indicated that they will be fearless and hopeful about their condition, 29% indicated that they will be surprised and sad, whereas 25% indicated that they will be ashamed and embarrassed if they were to be diagnosed with Drug Resistant TB. The results in Table 3 also show that 40% of the respondents indicated that they will talk or speak to no one, 37% of respondents indicated that they will tell or speak to their family friends and neighbors compared, whereas 23% of the respondents indicated that they will speak or tell the healthcare workers about their condition. The results also illustrate that when asked about what they will do if they suspect having Drug Resistant TB, 14% indicated that they will consult a herbalist, 12% initiate self-treatment, 7% will stay home rest and pray. Table 3 also shows that half of the respondents stated that they will go to the hospital as soon as they realize that they have TB. Almost a quarter indicated that they will only go when self-treatment does not work while others indicated that they will never go to the clinic. The results in Table 3 also show that the majority of the respondents believed that Drug Resistant TB infected should be forcefully isolated prevent its spread. The study also revealed that the 73% of the respondents believed that Drug Resistant TB infected people also deserve respect and fair treatment like every citizen. Table 3 further indicates that 68% respondents believed that people who default TB treatment should not be blamed for the spread of Drug Resistant TB. Table 3 further illustrate that the 78% of the respondents believed that it is not a waste of money to treat Drug Resistant TB infected people and that its spread can be curbed. The results in Table 3 also reveal that almost half of the respondents indicated that they do mind Drug Resistant TB infection as long as they were going to get disability grant. Lastly, the results in Table 3 indicate that 93% of the respondents knew that Drug Resistant TB is not only a disease of the poor and the HIV and AIDS infected people.

### 4.5. Participant's Drug Resistant TB Prevention Practices

Table 4 provides information related to respondents Drug Resistant TB prevention practices. The results in Table 4 show that the majority of the respondents knew that covering their mouth, when coughing and sneezing (85%), correct disposal of sputum (86%), avoidance of spitting indiscriminately (84%), wearing of protective mask (87%), and staying in well-ventilated areas or avoidance of close contact for long periods with infected people (79%) are Drug Resistant TB prevention practices.

This study also revealed that the only 52% of the respondents knew that taking of prescribed medication only when feeling sick and stopping it when feeling better is not an acceptable Drug Resistant TB prevention practice. The research further indicates that 79% of the respondents knew that performing traditional rituals and constantly praying were not a Drug Resistant TB prevention practice. The results further revealed that only 48% of the respondents knew that avoiding shaking hand with Drug Resistant TB infected people is not a preventative practice against the transmission and spread of Drug Resistant TB. The results also revealed that only 39% of the respondents knew that avoiding sharing of dishes, cups, and linen with the infected is not a preventative practice against the spread and transmission of Drug Resistant TB. Table 4 also indicates that 86% of the respondents knew that taking Drug Resistant TB drugs with the assistance of DOT supports or volunteers assisted in the management, prevention of the transmission and spread of Drug Resistant TB. The results further show that 80% and 84% of the respondents respectively, knew that constantly taking traditional herbs or medication and drinking water from spiritual and faith healers were not prevention practices towards the spread and transmission of Drug Resistant TB. Lastly, the research results in Table 4 reveal that 58% of the respondents knew that forceful isolation of Drug Resistant TB infected was not an acceptable and legal way of managing and preventing transmission and the spread of the disease in South Africa.

### 4.6. Participant's Drug Resistant TB Knowledge, Attitude, and Prevention Practices Scores

The results in Table 5 show poor knowledge, poor attitudes but good prevention practices among the research respondents.

### 4.7. Correlation between Participant's Knowledge, Attitudes and Prevention Practices

The results in Table 6 show a statistically significantly association between knowledge and attitudes (Pearson chi square *p* = <0.000). The results further show no statistically significant association between knowledge and practices and attitudes and practices respectively (Pearson chi square *p* = 0.120 and 0.136).

## 5. Discussion

The emergence of Drug Resistant strains of Mycobacterium tuberculosis has become a major threat to public health globally. South Africa's ability to meet the Sustainable Development Goals of ending TB by 2030, and WHO target of achieving a 100% decline in incidence and TB related deaths by 2035 are also threatened by the outbreak of Drug Resistant Tuberculosis. If the above targets are to be met, community members must possess appropriate knowledge with regard to the causes of MDR-TB, its sign and symptoms, transmission, prevention and treatment modes and methods so that they can take appropriate actions to control and prevent the spread of this disease. This study revealed poor knowledge, unfavourable attitudes but good prevention practices of Drug Resistant TB among some community members. This study further revealed statistically significant association between knowledge and attitudes (*p* = <0.001) even though no association was found between knowledge and practices (*p* = 0.120) and attitudes and practices (*p* = 0.136). These findings are similar to those of the previous studies [17–19]. These results show that Drug Resistant TB is still a phenomenon, which is not yet fully understood among communities, and it might be an indication of the fact that the current health education interventions on TB are not enough or addressing the target population. These findings, therefore, justify the intensification of health awareness and educational intervention, in order to address in the observed gap in knowledge, misconceptions, and erroneous beliefs that exist among community members.

This study found that the respondents had high knowledge about general issues relating to the severity, risk and perceptions on the causes of Drug Resistant Tuberculosis. These findings were similar to those of the study conducted in Grahamstown in the Eastern Cape Province of South Africa [19]. This also found that only 31% of the respondents correctly indicated that dust, smoking, drinking dirty water and poverty are not the causes of Drug Resistant Tuberculosis, which is consistent with the findings of previous studies [20] that have also revealed limited knowledge among regarding causes of Drug Resistant TB. The results also show that only two-thirds of the participants knew that incorrect and incomplete treatment regimen were also the causes of Drug Resistant Tuberculosis. Poor awareness regarding the aetiology of the disease may have a negative impact on community member's attitude towards health-seeking behaviour and preventative methods, as most people who have various beliefs and misconceptions may not visit health care facilities but opt for either traditional or spiritual alternatives [11]. The knowledge about the risk, perception about severity, and cause of a disease is an important aspect, which affects or influence health seeking behaviour and practices. The success of the Tuberculosis control program in the Nelson Mandela Metropolitan Municipality relies heavily on intensification of educational interventions and increased awareness and knowledge levels at community levels. These findings are an indication of a need for an intensive tuberculosis health educational intervention to address this knowledge gap.

Research shows that knowledge of signs and symptoms of a disease has a direct relationship with early presentation for diagnosis and treatment, which in turn affects prognosis or prediction. The results showed that the majority of the respondents had good knowledge of signs and symptoms of Drug Resistant TB as they cited weight loss, cough for more than three weeks chest pains and shortness of breath as its signs and symptoms, whereas a three-quarters of the respondents knew that nightmares and hallucinations were not. The research results also showed that all the respondents knew that coughing up of blood was a sign and symptom of TB, and these findings were similar to those of earlier studies by [21, 22].

The results showed that the respondents had poor knowledge and awareness of the Drug Resistant TB transmission modes and methods. The results of this study also showed that the majority of the respondents knew that Drug Resistant TB is transmitted through the air, when the infected person coughs or sneezes. These findings were similar to those of the previous studies by [11, 21, 24]. The findings also showed that some of the respondents did not know that Drug Resistant TB was not a hereditary disease spread through blood, and transmitted through, witchcraft, and these findings were consistent with those of the previous studies [10, 24, 25]. These results clearly illustrate that a significant proportion of the study participants still lack general knowledge about certain aspects of the disease and this is detrimental to the success of the TB control program. The study also found that the respondents knew that Drug Resistant TB was not transmitted through hugs and handshake with the infected person and touching of items in public spaces or areas. These findings were similar to those of previous studies [11] but in contrast to [25]. This has important implication for the TB program in the area, and if not addressed quickly, it has a potential of increasing diagnosis and treatment delays as well as exacerbating the spread of the disease. Knowledge on prevention modes and methods of the disease is another important factor in disease prevention and control program.

The results indicate that, the majority of the respondents in the present study knew that Drug Resistant TB treatment was provided freely at designated Drug Resistant TB centres. 73% of the respondents knew that Drug Resistant TB is not infectious while on treatment and after completion of treatment and that 88% also knew that Drug Resistant TB treatment outcomes are evaluated through sputum tests and chest X-rays. These findings are similar to those of the study conducted by [23], where a majority of respondents indicated that chest X-rays was the diagnostic tool for TB and that sputum examination and chest X-rays were used to diagnose and evaluate outcomes for TB. This study also found that only a third of the respondents knew the duration of treatment and that psychosis, hearing and vision loss are side effects of Drug Resistant TB treatment. These findings were in conflict to those of [26, 27], where it was found that of the majority of respondents knew the duration of treatment and that hearing loss or deafness and psychosis are the side effects of the Drug Resistant Tuberculosis drugs or treatment. In this study, the respondents still lacked some important knowledge concerning side effects treatment and diagnostic methods against Drug Resistant Tuberculosis infection. This has important implication for the TB prevention and control program in the area in that if people are not aware of these issues, they may delay health seeking and default treatment once they feel better or when they suffer from treatment side effects. This makes them vulnerable to relapse or re-infection hence increasing the possibility of the spread of Drug Resistant Tuberculosis.

The results of this study showed that respondents had unfavourable or negative attitudes about Drug Resistant TB. Cultural beliefs, value and norms affect health-seeking behaviour. Social isolation, prejudice, and negative attitudes or views that people have about TB make people hide and avoid seeking medical assistance early enough [28]. The results obtained from this study illustrate that when respondents were asked about the reaction of community members if they had Drug Resistant TB, the majority of the respondents indicated that the community will avoid them but remain friendly, 27% indicated that they will be supportive and help, whereas 23% believed that they will be rejected. The above mentioned findings are similar to those found in a study conducted by [23]. The study also found that when respondents were asked about how they will react if they were told that they had Drug Resistant TB, 46% indicated that they will be fearless and hopeful, 29% surprised and 25% embarrassed and ashamed. The study also found that when asked about whom they would talk to about their Drug Resistant TB diagnosis or infection, the majority of the respondents indicated that they will speak to no one, 37% with family friends and neighbors, while only 23% indicated that they would speak to health care workers. The findings of this study are consistent with those of a study conducted in Venda Limpopo Province of South Africa, where it was found that people hided or did not disclose their status for fear of isolation [29, 30]. People infected with Drug Resistant TB often endure pain and suffering not only physically but also emotional distress that results from stigma imposed on them by society, isolation and rejection by community, friends, and family members.

The study found that more than a third of research respondents believed that Drug Resistant TB was transmitted through witchcraft, 20% indicated that they will constantly take traditional herbs and perform traditional rituals to manage and prevent infection with Drug Resistant TB. These findings were consistent with those of previous studies [22, 31]. The results of the present study also confirm the use and existence of dual health systems (African or traditional and modern or western). The results of the present study further highlight the importance of fostering collaborations with other providers such as traditional and spiritual healers in TB care. In this study when respondents were asked about what they will do if they suspect Drug Resistant TB, 14% stated that they will consult herbalist, 7% stated that they will stay home, rest and pray, while 12% initiated self-treatment. These findings are similar to those of earlier studies by [23], but in contrast to [11].

Family members of the uncured XDR-TB patients indicated that they will not get XDR-TB because they believed that God was protecting them [32]. This further reveals that some of the respondents have faith in spiritual powers and believed that by praying they will be protected from Drug Resistant TB infection. These findings highlight misconceptions and erroneous beliefs that community members have with regard Drug Resistant TB. These beliefs and practices need to be properly addressed in collaboration with the different ministries as they might lead to complete disregard of prevention practices against Drug Resistant TB.

This study also found that 23% of the respondents stated that they will go to a clinic or hospital when self-treatment does not work, while 27% indicated that they will never seek medical assistance. The majority of the respondents indicated that they will visit healthcare facilities as soon as they realise they have TB, and that is similar to the findings of previous studies [11, 23]. Ignorance, lack of knowledge, stigmatization, misconceptions, and erroneous beliefs are issues that hinder successful TB control initiatives [33, 34]. TB suspects usually presented themselves late to seek medical assistance because they first try to seek help from traditional healers, because they believed that TB was caused by witchcraft, ‘evil eye' and Satan [35]. The study also clearly illustrates that a significant proportion of the study participants still lack general knowledge about certain aspects of the disease. The above results further show that community members had distorted information about Drug Resistant TB, and that some of the respondents prefer using other alternatives rather than public health institutions. Such preferences might be due to lack of confidence and mistrust with public health institutions, and that might affect negatively on TB control program and the continuity of care. In a study conducted in South Africa by [36] it was found that the stigma and discrimination associated with TB in both urban and rural communities played an important role in the spread and control of the TB epidemic. The concept of blame is a crucial determinant for stigma and discrimination to exist. This study found that less than half of the respondents believed that it was incorrect to forcefully isolate Drug resistant TB infected people. This study also found that a quarter of the respondents believed that Drug Resistant TB infected people do not deserved respect and a fair treatment like everyone else. A third and 22% of the respondents, respectively, believed that it was okay to discriminate and treat badly Drug Resistant TB infected people and that they should be blamed for spreading the disease and this was consistent with the findings of an earlier study conducted in Grahamstown Eastern Cape Province of South Africa [19, 37]. The above results highlight the fact that a number of the respondents in the present study had disregard the basic human rights that are enshrined in the South African Constitution and other pieces of legislation that are governing management and control of Tuberculosis in South Africa. The results also highlight the need for intensification of health education interventions that are not only aimed at increasing the general knowledge but also the (human rights) legal and ethical issues related to the management and control of the Drug Resistant TB.

The study also found that some of the respondents believed that it was a waste of money to treat Drug Resistant TB infected people as it was not curable and its spread could not be prevented. These perceptions among some of the research respondents could possibly be due to the extensive media coverage of the disease that might have perpetuated a fear among South Africans about the severity of the disease if it was left untreated during early years of the breakout in the KwaZulu-Natal Province where many people died [38]. Infection prevention practices such as isolation of patients and wearing of mask and other protective clothing when health care workers are dealing with TB or Drug Resistant TB infected people might also have raised fears and created confusion among community members, regarding its severity, transmissibility and curability [39, 40, 41]. Statements that Drug Resistant TB patients were highly infectious and a danger to society, which were issued in 2008 when patients used their illness to scare off security guards and fled from an MDR-TB hospital in the Eastern Cape, might have influenced perceptions and attitudes of the respondents towards Drug Resistant TB [39, 40]. The number of people who are dying while on Drug Resistant TB treatment, due to late diagnosis of the disease, diagnostic, technological and infrastructural challenges, coinfection with HIV and AIDS and treatment default, might also be the reasons for the perception that it was a waste of money to treat Drug Resistant TB infected as it was not curable [41].

This study also found that despite the high perceived severity among respondents about Drug Resistant TB and the lethal nature of the Drug Resistant TB if left unattended, almost half of the research respondents indicated that they did not mind Drug Resistant TB infection. These findings highlight the socio economic plight of some of the research respondents as some regarded TB infection as means to access some form of income in the form of disability grant. [42] found that some people deliberately get infected with TB so that they can receive temporary grant and it acts as a perverse incentive. These findings highlight some of the challenges for the TB control program and further highlight the need for further research into this area. This study also found that 7% of the respondents believed that Drug Resistant TB was a disease of the poor and HIV and AIDS infected people.

These findings were similar to those of the previous study by [43] who found that TB was stigmatized and associated with poverty, dirt, poor nutrition and bad health practices such as smoking. Thus, these social factors are associated with perpetuation of stigma and discrimination of the disease. The negative judgmental attitudes towards TB need to be removed so that there can be better TB control to reduce death from TB and the spread of Drug Resistant TB. The results of this study highlight a need for the extension of coverage and intensification of health education interventions that are aimed at reducing stigma and increasing knowledge and awareness.

The results illustrate that the majority (59%) of the respondents had good knowledge of Drug Resistant TB prevention practices. The findings of this study are similar to those of the studies that were conducted by [22, 23] where the study participants had good knowledge of prevention practices towards TB. The study found that (85%) said through covering of mouth and nose when the infected person is coughing and sneezing, (86%) correct disposal of sputum, (84%) by not spitting indiscriminately, and lastly (79%) by staying in a well-ventilated area and avoiding close contact for long period with infected people as ways of preventing spread and infection with Drug Resistant TB. These findings are similar to those of previous studies by [25, 26, 44]. Although the majority of the respondents knew prevention practices of Drug Resistant TB, it is also of significance to note that some of the respondents in this study had low perceived susceptibility, poor knowledge and awareness levels regarding these prevention practices, and thereby at risk of infection with the disease. Only 48% of the respondents knew that avoiding hugging and shaking hand with Drug Resistant TB infected is not a preventive practice against its transmission. These findings were similar to those of a previous study by [24]. Similar results were found in previous studies by [10, 45] where more than a third of the respondents believed that MDR-TB was transmitted and caused by kissing and touching infected people and therefore avoid kissing and touching the infected persons were regarded as means of preventing infection and its spread. The results show that in the present study, (39%) of the respondents knew that avoiding sharing of dishes, cups, and linen with the infected is not a preventative practice against the spread and transmission of Drug Resistant TB. This is consistent with the findings of a study by [23]. The findings of the present study also revealed that about 52% of the respondents knew that taking prescribed medication only when feeling sick and stopping it when feeling better is not an acceptable Drug Resistant TB prevention practice. A study conducted to assess patient's knowledge and attitudes of MDR-TB in Kwa-Zulu-Natal, SA revealed that 6.1% of the respondents would stop their medication when they felt better and another 4.2% stated that they would stop the medication if they felt worse [10, 30].

The research findings indicate that 21% of the respondents did not know that performing traditional rituals is not an acceptable Drug Resistant TB prevention practice. The results further show that 20% of the research subjects believed that constantly taking traditional or herbal remedies was an acceptable prevention practice towards the spread and transmission of Drug Resistant TB. These findings are similar to those of the previous studies where respondents indicated using herbal remedies as treatment and prevention method for TB [11]. Such practices might be promoted by beliefs such as those that were found among the Massai in Tanzania where respondents believed that traditional healers were capable of curing TB [46]. The results also showed that 22% and 16% of the respondents believed that by constantly praying and drinking water from faith and spiritual healers would prevent them from Drug Resistant TB infection, respectively. These findings were similar to those of a study conducted by [32] where family members of the uncured XDR-TB patients indicated that they will not get XDR-TB because they believed that God was protecting them. These findings reveal that some of the respondents have faith in spiritual powers and believed that by praying they will be protected from Drug Resistant TB infection. These findings also highlight misconceptions and erroneous beliefs that community members have with regard to Drug Resistant TB. These beliefs and practices need to be addressed in collaboration with the different ministries as they might lead to complete disregard of prevention practices against Drug Resistant TB, and thereby exacerbate its spread.

The result of the current study illustrates that some of the respondents have poor knowledge and awareness levels, negative attitudes, and poor prevention practices and thereby at risk of contracting Drug Resistant TB. Knowledge influences attitudes and health-seeking behavior or practices. If incorrect information, misconception, and erroneous beliefs are held about a disease, people will not be able to recognize the signs and symptoms of the disease, and thereby delay seeking help, and that will affect negatively on health seeking and TB control [47]. The observed gap in knowledge and awareness, poor attitudes, and prevention practices among some of the research respondents in this study generally shows health risk. These findings can motivate the Nelson Mandela Health District and the Eastern Cape Provincial Department of Health to collaborate with NGO and other NPO to scale up educational interventions, awareness campaigns, and outreach program on prevention, transmission, diagnosis, treatment, and management of Drug Resistant TB in the municipality and the province as a whole.

### 6. Limitations

The study was conducted in a limited area in Port Elizabeth, in the NMBM, and therefore generalization of the findings to provincial, national, and international settings is limited. Secondly, there were limitations in making inferences about the meaning of the research results because this study was descriptive and cross sectional in nature. Thirdly, since the sample was not chosen at random, but through convenience sampling, and therefore the sample was not representative of the population that was being studied. Lastly, data collected were based on self-reporting.

### 7. Recommendations

Based on the findings of this study, it is recommended that Drug Resistant TB health education interventions and awareness campaigns are intensified. Future education interventions should focus addressing misconceptions and erroneous beliefs about Drug Resistant Tuberculosis. Health promotion initiatives and program coverage should be extended to include places where most people spend most of their time (schools, shopping malls, churches, and sports clubs). In delivering such programs, health educators together with People living with Drug Resistant TB should be used to facilitate the learning. They should be offered opportunities to interact, and offer first-hand information about their lived experience, as such interaction could assist in increasing the general awareness and knowledge levels about different types of TB and also in creating cues for action and promote adaption of positive health behaviours and actions, and thereby assist to curb the spread of Drug Resistant Tuberculosis. Close collaboration needs to be established among community leaders, spiritual leaders of various ministries, traditional health practitioners, and NGO and government. Health educators can also work together with community leaders, spiritual or faith and traditional health practitioners, Healthcare workers (HCW), and Ward based outreach team (WBHOT) in the dissemination of knowledge about Drug Resistant TB in the Nelson Mandela Bay Municipality and the Eastern Cape Province at large.

### 8. Conclusion

This research assessed knowledge, attitudes, and practices Drug Resistant TB among community members in Port Elizabeth residents. The pattern of respondent's responses was shown using descriptive statistics. In this study, the majority of respondents had poor knowledge and unfavourable attitudes, but good prevention practices of Multi Drug Resistant Tuberculosis. The majority of the respondents are considered to be at high risk and vulnerable to infection. Some of the respondents are also highly susceptible to Drug Resistant TB infection because of their socio-economic conditions. Furthermore, the results indicate gaps in knowledge about causes, transmission, prevention, treatment modes, and methods of Drug Resistant TB, which could make them vulnerable to Drug Resistant TB infection. These findings highlight the need for intensification of health education interventions about Drug Resistant TB treatment and its side effects. Inadequate knowledge and misconceptions about prevention and transmission of Drug Resistant TB are a threat to the successful control and prevention of the spread of this deadly but preventable disease. Coupled with access to healthcare and sound financial and physical resources at population level, the success of the TB Control Program also depends on community members having appropriate knowledge, nondiscriminatory or judgemental attitudes towards the Drug Resistant TB infected, and using proper methods to prevent the spread of the disease. Drug Resistant TB information and programs that are culturally sensitive should be provided to community members using different media or methods in order to enlighten community members and dispel misconceptions and erroneous beliefs with regard to Drug Resistant TB. The efforts of the TB control program will be fruitless if community misconceptions, erroneous beliefs about the risk, causes, transmission, prevention, and management of Drug Resistant TB are not addressed.

## Figures and Tables

**Table 1:** Socio-demographic characteristics of the participants.

Variables	*N*(400)					
	Mean	Std. dev	Min	Max

Age	38	12.2	18	68

	Frequency (n)	Percentage (%)		
*Gender*				
Male	190	47.50		
Female	210	52.50		
*Marital status*				
Single	218	54.50		
Married	114	28.50		
Divorced	20	5.00		
Widow	48	12.00		
*Race*				
African	270	67.50		
Coloured	93	23.25		
Indian	12	3.00		
White	25	6.25		
*Educational status*				
No formal education	41	10.25		
Primary education	150	37.50		
High school education	86	21.50		
Tertiary education	123	30.75		
*Occupational status*				
Employed	150	37.50		
Unemployed	250	62.50		
*Family monthly income*				
R 4999 and less	233	58%		
R 5000–R9999	71	18%		
R 10000–R14999	61	15%		
R 15000 and up	35	9%		

Fana, Ijeoma and Eyles: 2018.

**Table 2:** Sources of Drug Resistant Tuberculosis information.

Variables	*N* (400)	
	Frequency (n)	Percentage (%)
Family and friends	86	21.50
TV and radio	137	34.25
TV, radio, and internet	121	30.25
Teachers and healthcare workers	56	14.00

Fana, Ijeoma and Eyles: 2018.

**Table 3:** Participant's responses on perceptions and attitudes about Drug Resistant TB.

Statements about community perceptions and attitudes of DR-TB	*N* = 400	
	Freq.	%
*3.1. What will be the community reaction if you have Drug Resistant TB?*		
a. They will reject you	92	23
b. They will be avoid you but remain friendly	201	50
c. They will be supportive and helpful	107	27
*3.2. How will you react if you told you have Drug Resistant TB?*		
a. I will be fearless and hopeful	183	46
b. I will be ashamed and embarrassed	100	25
c. I will be surprised and sad	117	29
*3.3. Who will you tell or speak to if you have Drug Resistant TB?*		
a. Friends, family members, and neighbors	149	37
b. Health care workers	93	23
c. No one	158	40
*3.4. What will you do if you suspect you have Drug Resistant TB?*		
a. Consult a herbalist	56	14
b. Visit health care centre or hospital	268	67
c. Just stay at home, rest and pray	29	7
d. Initiate self-treatment	47	12
*3.5. When will you go to a clinic or hospital after getting TB?*		
a. When self-treatment does not work	92	23
b. As soon as I realise that I have TB	201	50
c. Never	107	27
*3.6. Community attitudes and beliefs about Drug Resistant TB*		
a. Forcefully isolation of Drug Resistant TB infected is correct to prevent its spread	188	47
b. Drug Resistant TB infected deserves respect and fair treatment like everyone else	290	73
c. Treating Drug Resistant TB infected people is a waste of money	313	78
d. Those who default treatment are to be blamed for the spread of Drug Resistant TB	273	68
e. I do not mind Drug Resistant TB infection as long as I will get disability grant	204	51
f. Drug Resistant TB is a disease of the poor and HIV infected	370	93

**Table 4:** Participants responses to Drug Resistant TB prevention practices.

Statements relating to prevention practices of Drug Resistant TB	*N* = 400	
	Freq.	%
1. Covering my mouth when coughing and sneezing	338	85
2. By taking prescribed medication only when feeling sick	206	52
3. Disposing sputum correctly regularly	345	86
4. Performing traditional rituals	316	79
5. Stay in a ventilated area and avoid close contact with infected	316	79
6. Not spitting indiscriminately	336	84
7. Avoiding shaking hands with DR-TB infected people	191	48
8. Constantly taking traditional medication or herbs	319	80
9. Constantly praying	313	78
10. Taking prescribed DR-TB drugs with assistance of DOT supporter	338	85
11. Avoid contact with infected person even if he is on treatment or cured	338	85
12. Avoid sharing dishes, cups and linen with the infected people	155	39
13. Wearing an N 95 protective or surgical mask	347	87
14. Drinking water from faith and spiritual healers	335	84
15. Forcefully isolating Drug Resistant TB infected	232	58

**Table 5:** Respondents knowledge, attitudes, and prevention practices scores.

Variables	*N* (400)	
	Frequency (*n*)	Percentage (%)
*Knowledge*		
Good	214	56.00
Poor	176	44.00
*Attitude*		
Good/favourable	94	23.50
Poor/unfavourable	306	76.50
*Practices*		
Good	235	58.75
Poor	165	41.25

**Table 6:** Association between knowledge and attitudes, knowledge and practices and attitude and practices.

Knowledge	Attitudes		
	Yes	No	Total
Yes	88	88	176
No	6	218	224
	94	306	400
Pearson chi square *p* = <0.001			

Knowledge	Practices		

	Yes	No	Total
Yes	111	65	176
No	124	100	224
	235	165	400
Pearson chi square *p* = 0.120			

Attitudes	Practices		

	Yes	No	Total
Yes	49	45	94
No	186	120	306
	235	165	400
Pearson chi square *p* = 0.136			

## Data Availability

The data used in this study can be provided by the author upon request.
